# Analgesic efficacy of continuous serratus anterior plane block versus intercostal nerve block and their combination in VATS lobectomy: results from a prospective randomized trial

**DOI:** 10.3389/fsurg.2025.1607150

**Published:** 2025-05-27

**Authors:** Hyun Ah Lim, Gongmin Rim, Kwanyong Hyun, Yong Jin Chang, Deog Gon Cho

**Affiliations:** ^1^ Department of Thoracic and Cardiovascular Surgery, The Catholic University of Korea Seoul St. Mary’s Hospital, Seoul, Republic of Korea; ^2^ Department of Thoracic and Cardiovascular Surgery, CHA Bundang Medical Center, CHA University School of Medicine, Seongnam, Republic of Korea; ^3^ Department of Thoracic and Cardiovascular Surgery, The Catholic University of Korea St. Vincent’s Hospital, Suwon, Republic of Korea

**Keywords:** video-assisted thoracoscopic surgery, lobectomy, serratus anterior plane block (SAPB), intercostal nerve block (INB), pain control

## Abstract

**Background:**

Effective postoperative pain management following video-assisted thoracoscopic surgery (VATS) lobectomy is essential to optimize recovery and minimize opioid consumption. This study aimed to compare the analgesic efficacy of ultrasound-guided continuous serratus anterior plane block (SAPB), intercostal nerve block (INB), and their combination (SAPB + INB) in patients undergoing VATS lobectomy.

**Methods:**

In this single-center, single-blinded, randomized controlled trial, 90 patients undergoing VATS lobectomy for confirmed or suspected lung cancer were randomly assigned to one of three groups: INB (*n* = 30), continu.ous SAPB (*n* = 30), or SAPB + INB (*n* = 30). The primary outcome was postoperative pain assessed using the visual analog scale (VAS) at 24 h. Secondary outcomes included VAS scores at 1, 3, 6, 12, 48, and 72 h postoperatively, cumulative opioid consumption, length of hospital stay, and postoperative complications.

**Results:**

No significant differences in VAS scores were observed among the three groups at 24 h postoperatively. All groups maintained acceptable pain levels (VAS < 4) throughout the study. However, opioid consumption was significantly lower in both the SAPB and SAPB + INB groups compared to the INB group at all time points (*p* < 0.01).

**Conclusions:**

Continuous SAPB, INB, and SAPB + INB were all effective for postoperative pain management after VATS lobectomy. However, INB alone was associated with significantly higher opioid use. Given its technical simplicity, prolonged analgesic effect, and opioid-sparing properties, continuous SAPB represents a valuable component of multimodal analgesia in enhanced recovery protocols.

**Clinical Trial Registration:**

Identifier KCT0009683.

## Introduction

Video-assisted thoracoscopic surgery (VATS) lobectomy is a minimally invasive procedure that reduces surgical trauma compared to thoracotomy (1, 2). Although VATS is associated with less postoperative pain and faster recovery than conventional thoracotomy, optimal postoperative pain management remains crucial for enhancing recovery, facilitating early mobilization, and reducing pulmonary complications (3). Enhanced Recovery After Surgery (ERAS) protocols for thoracic surgery emphasize multimodal analgesia, incorporating regional anesthesia and local anesthetic techniques to minimize opioid consumption (4, 5). Thoracic paravertebral block (PVB) and thoracic epidural anesthesia (TEA) are commonly preferred for pain control; however, they have limitations, including potential neurologic complications and the need for an anesthesiologist (6, 7).

Intercostal nerve block (INB) and serratus anterior plane block (SAPB) have emerged as simpler and safer alternatives. prominence. SAPB, which targets the lateral cutaneous branches of the intercostal nerves (T2–T9), provides effective chest wall analgesia (8). When administered as a continuous infusion, SAPB offers prolonged pain relief and reduced opioid consumption. INB is also widely utilized and has been demonstrated to be effective in reducing postoperative pain. Additionally, both INB and SAPB are technically straightforward procedures that can be easily performed by surgeons, with proven efficacy and safety. Despite the increasing use of SAPB and INB, limited data directly compare continuous SAPB, INB, and their combination (SAPB + INB) for VATS lobectomy. This study aims to evaluate the efficacy of continuous SAPB, INB, and their combination as potential alternatives to current regional anesthesia techniques. Specifically, the study investigates their effects on postoperative analgesia, opioid requirements, and overall recovery profiles following VATS lobectomy.

## Materials and methods

### Study design

**Figure 1:**
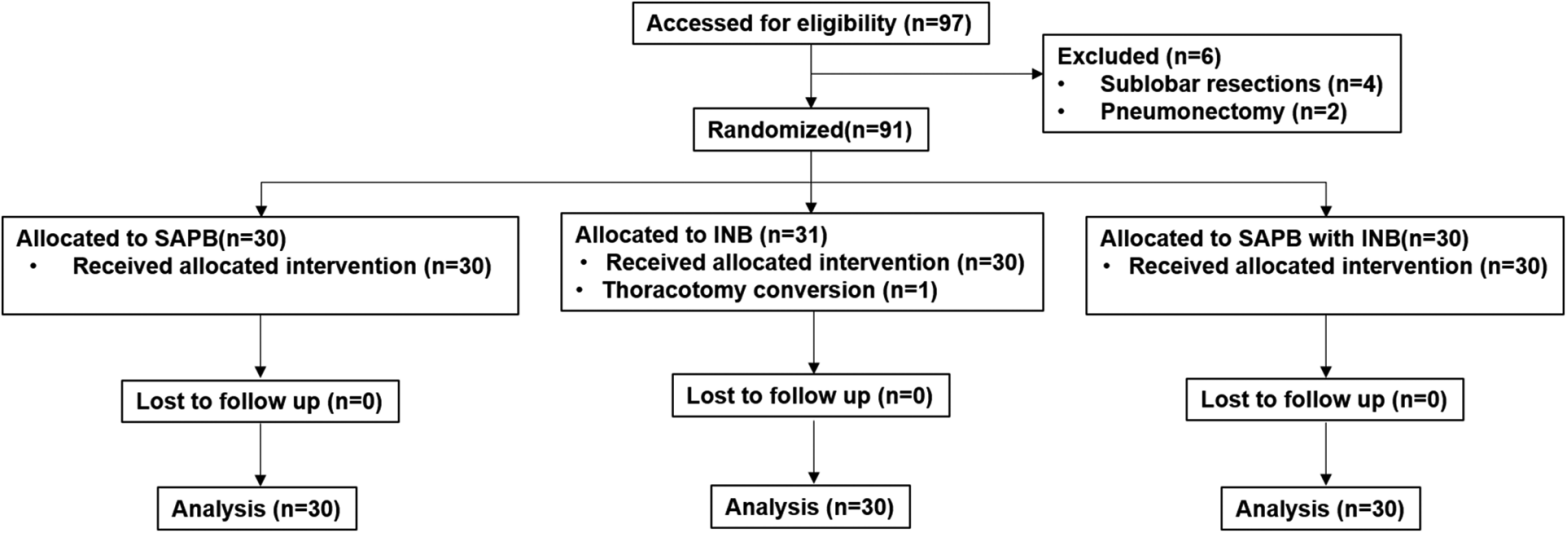
CONSORT flow diagram of the study.

This randomized, prospective, 3-arm, single-blinded controlled trial was conducted at a single center from August 2024 to December 2024. The Institutional Review Board approved and continuously monitored this study. (reference number: VC24EISI0089), and registered at Clinical Research Information Service on August 6th, 2024 (reference number: KCT0009683). Written informed consent was obtained from all eligible patients prior to enrollment. Ninety patients undergoing VATS lobectomy for primary lung cancer were randomized into three groups: (1) INB group (*n* = 30, Group I) (2) SAPB group (*n* = 30, Group S) (3) SAPB + INB group (*n* = 30, Group H) (Figure 1). All procedures was conducted in accordance with the Declaration of Helsinki throughout the study period.

The primary outcome was postoperative pain intensity assessed by the Visual Analog Scale (VAS) at 24 h after surgery. Secondary outcomes included VAS scores at 1, 3, 6, 12, 48, and 72 h postoperatively, total intravenous (IV) rescue analgesic consumption, length of hospital stay, total operative time, chest tube duration, and postoperative complications such as pneumonia, bleeding requiring transfusion, and pleural effusion.

### Patient selection

This study was a three-arm parallel trial with an allocation ratio of 1:1:1, enrolling a total of 90 patients. Inclusion criteria included patients of any sex, aged 18–75 years, with an American Society of Anesthesiologists (ASA) physical status classification of I to III, who were scheduled to undergo VATS lobectomy at St. Vincent's Hospital for confirmed or suspected lung cancer. Exclusion criteria were as follows: known allergy to local anesthetic agents, significant coagulopathy, history of prior ipsilateral thoracic surgery, chronic pain conditions or psychiatric disorders, receipt of preoperative chemotherapy or radiotherapy, difficulty in communicating with the study team, concurrent participation in other clinical trials, undergoing surgical procedures other than lobectomy (e.g., sublobar resection or pneumonectomy), and refusal to participate. A flow diagram summarizing patient enrollment and allocation is presented in Figure 1.

### Randomization

A computer-generated individual randomization unit was employed, with a non-stratified sequence in four blocks. Once consent for study participation was approved, a sequentially numbered opaque envelope was accessed to obtain the next allotment. All data were analyzed on an intention-to-treat basis, and patients remained in their assigned groups.

### Basal pain management protocol

All patients received standardized anesthesia and postoperative analgesia according to the institutional protocol. General anesthesia was induced using IV lidocaine (1–2 mg/kg, maximum 100 mg) and propofol (2–4 mg/kg, maximum 150 mg), with rocuronium (0.6–1 mg/kg, maximum 60 mg) or vecuronium (0.1 mg/kg, maximum 10 mg) to facilitate endotracheal intubation. Routine postoperative IV ibuprofen (800 mg every 12 h until postoperative day two) was administered. IV patient-controlled analgesia (PCA) was omitted in all three groups to minimize opioid consumption and assess the efficacy of regional blocks. Postoperative oral analgesics included ibuprofen (400 mg every 8 h) and acetaminophen (650 mg every 8 h). For breakthrough pain, IV ketorolac (0.5 mg/kg every 6 h) and pethidine (0.5 mg/kg every 6 h) were administered as rescue analgesics on an as-needed basis. The total rescue analgesic consumption was determined by conversion to oral morphine milligram equivalent (MME). Pain assessments were made at 1, 3, 6, 12, 24, 48, and 72 h postoperatively by the study investigator, who was blinded to group assignment. Pain intensity was scored by VAS in the supine position (resting, VAS-R) and upright position when coughing (dynamic, VAS-D). We considered a VAS score <4 to be tolerable, determining the efficacy of pain control management.

### Statistical analysis

Normally distributed continuous variables were presented as means with standard deviations (SD), while non-normally distributed (skewed) data were expressed as medians with interquartile ranges (IQR). Categorical variables were summarized as frequencies and percentages. Normality was assessed using the Shapiro–Wilk test. Comparisons among the three study groups were performed using one-way analysis of variance (ANOVA) for normally distributed continuous variables and the Kruskal–Wallis test for non-parametric data. For categorical variables, the Chi-square test or Fisher's exact test was used as appropriate. *post-hoc* analysis was conducted with Bonferroni correction to adjust for multiple comparisons. All statistical analyses were performed using SPSS software (version 25.0; IBM Corp., Armonk, NY, USA), and a *p*-value of <0.05 was considered statistically significant.

### Continuous SAPB

**Figure 2:**
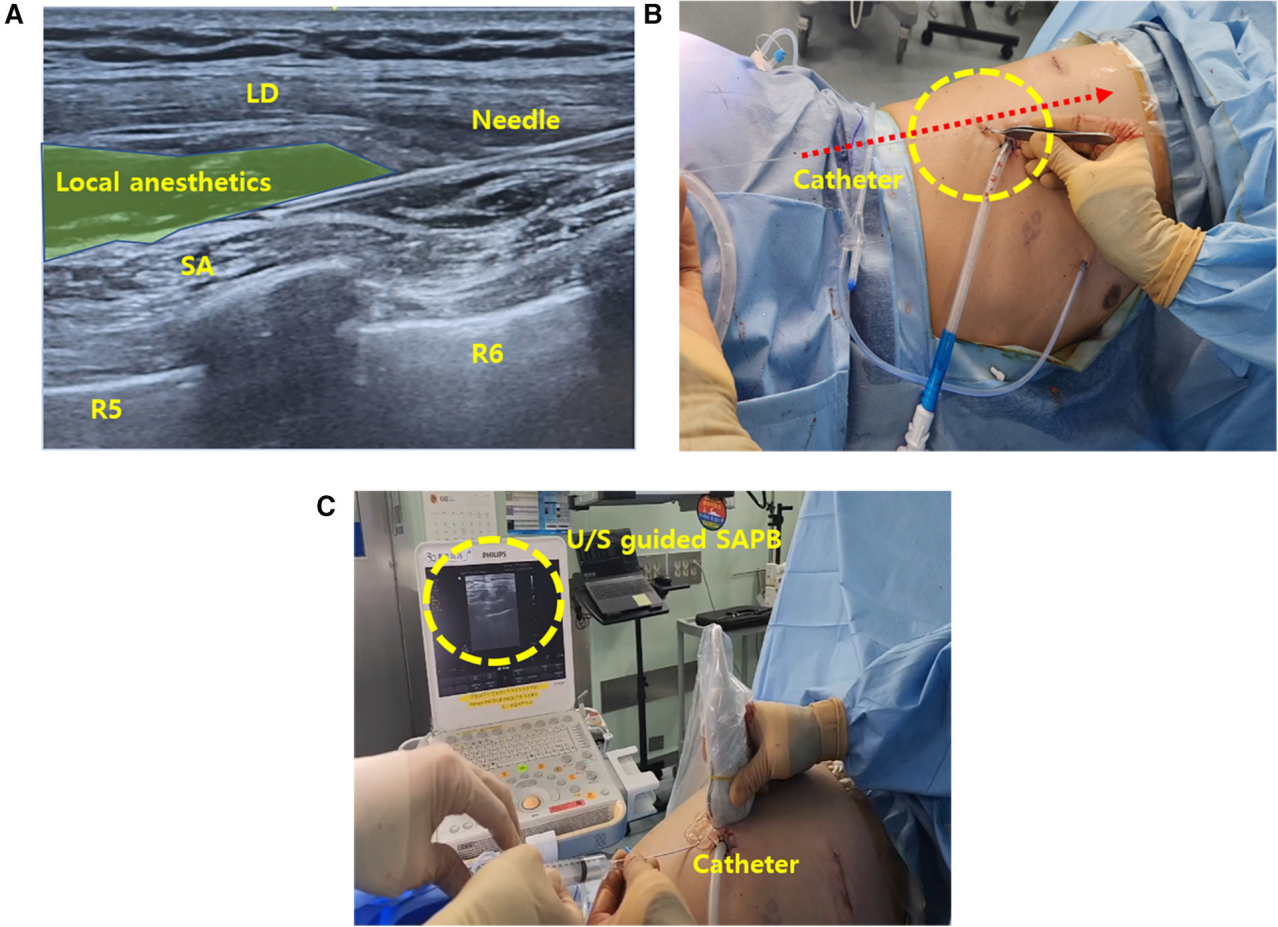
Intraoperative SAPB **(A)** illustration of ultrasound-guided SAPB, demonstrating the injection of local anesthetic between the SA and LD muscles. **(B)** Ultrasound image depicting needle placement and anesthetic spread within the interfascial plane. **(C)** Ultrasound-guided catheter placement into the serratus anterior plane for continuous regional analgesia. SAPB, serratus anterior plane block; U/S, ultrasound; SA, serratus anterior; LD, latissimus dorsi.

Following completion of surgery, the ultrasound-guided continuous SAPB was performed under strict aseptic conditions. With the patient in the lateral decubitus position, a high-frequency linear ultrasound transducer was placed at the midaxillary line at the level of the 7th–8th rib on the side of the block (Figure 2A) (8). After identifying the serratus anterior (SA) and latissimus dorsi (LD) muscles, an introducer needle (Painfusor, Baxter Inc., Deerfield, IL, USA) was inserted in a caudo-cranial direction under real-time ultrasound guidance using an in-plane technique. The needle was progressively advanced within the interfascial plane between the SA and LD muscles, and 35 ml of 0.3% ropivacaine was incrementally injected, with appropriate spread confirmed by ultrasound, allowing cranial diffusion toward the T4–T5 levels corresponding to the primary surgical site (Figure 2B) (9). For continuous SAPB, a 15 cm infusion catheter was placed into the serratus anterior plane under ultrasound guidance (Figure 2C). The catheter was anchored to the skin using a single 3-0 nylon suture and covered with a sterile transparent dressing. A Painfusor elastomeric infusion pump filled with 300 ml of 0.25% ropivacaine was connected to the catheter, delivering the anesthetic at a continuous flow rate of 5 ml/h. The catheter was removed on postoperative day 3.

### INB

Before closing the wound after the lobectomy, thoracoscopic-guided single-injection INB at T3–T9 with 2–3 ml of 0.25% ropivacaine per level.

### Continuous SAPB with INB

Combination of single-injection INB and continuous SAPB was performed. After INB, ultrasound guided SAPB was performed sequentially.

## Results

Between August and December 2024, a total of 97 patients were assessed for eligibility. After excluding 6 patients based on predefined criteria, 91 patients were randomized into three groups. One patient in the INB group was excluded from the final analysis due to intraoperative conversion to thoracotomy, resulting in a total of 90 patients included in the final analysis (Figure 1). There were no statistically significant differences among the three groups with respect to baseline demographic and clinical characteristics, including age (*p* = 0.35), body mass index (*p* = 0.40), sex distribution (*p* = 0.82), ASA classification (*p* = 0.84), or operation time (*p* = 0.19) (Table 1). Histopathological evaluations confirmed lung cancer diagnoses in all participants, with no significant differences in TNM stage distribution among the groups (*p* = 0.40) (Table 2).

**Table 1:** Baseline characteristics of study groups.

	INB (group I) (*n* = 30)	SAPB (group S) (*n* = 30)	INB with SAPB (group H) (*n* = 30)	*P* value*
Age, years	66.9 (±7.2)	65.5 (±10.2)	69.1 (±9.0)	0.35
Height, cm	161.1 (±7.7)	162.8 (±8.8)	160.1 (±9.5)	0.70
Weight, kg	60.1 (±9.1)	63.8 (±10.9)	62.4 (±11.2)	0.45
BMI, kg/m^2^	23.06 (±2.2)	23.9 (±2.7)	24.06 (±3.3)	0.40
Male sex (percentage)	14 (83.33)	15 (87.5)	13 (80.77)	0.82
Race, Asian	25 (100)	25 (100)	25 (100)	1.000
ASA class				0.84
ASA Ⅰ	2 (8)	24 (100)	24 (96)	
ASA Ⅱ	22 (88)		1 (4)	
ASA III	1 (4)			

Data expressed as mean (SD) or *n* (%).

INB, intercostal nerve block; SAPB, serratus anterior plane block; ASA, American Society of Anesthesiologists; BMI, body mass index; SD, standard deviation.

* Significance, *p* < 0.05.

**Table 2:** Perioperative characteristics of study groups (INB vs. SAPB vs. INB with SAPB).

	INB (group I) (*n* = 30)	SAPB (group S) (*n* = 30)	INB with SAPB (group H) (*n* = 30	*P* value*
Histopathology of lung cancer				0.55
Adenocarcinoma	21	24	21	
Squamous	3	1	2	
Others	1		2	
TNM stage (8th)				0.40
Ⅰ	19	18	17	
Ⅱ	2	5	4	
III	4	2	4	
Operation time, min	140.5 (±42.7)	144.6 (±46.1)	152.9 (43.8)	0.19
EBL, ml	148 (±151.1)	149.2 (±156.9)	115.2 (±193.4)	0.72
Length of stay, days	5.6 (±1.4)	5.6 (±1.1)	5.8 (±1.2)	0.65
CTD, days	3.2 (±46.1)	3.3 (±1.5)	3.4 (±1.8)	0.85
Complications				
Pneumonia	0	1 (4)	1 (4)	0.87
30day death	0	1 (4)	0	0.79

Data expressed as mean (range, SD) or *n* (%).

INB, intercostal nerve block; SAPB, serratus anterior plane block; EBL, estimated blood loss; CTD, chest tube duration; SD, standard deviation.

* Significance, *p* < 0.05.

**Figure 3:**
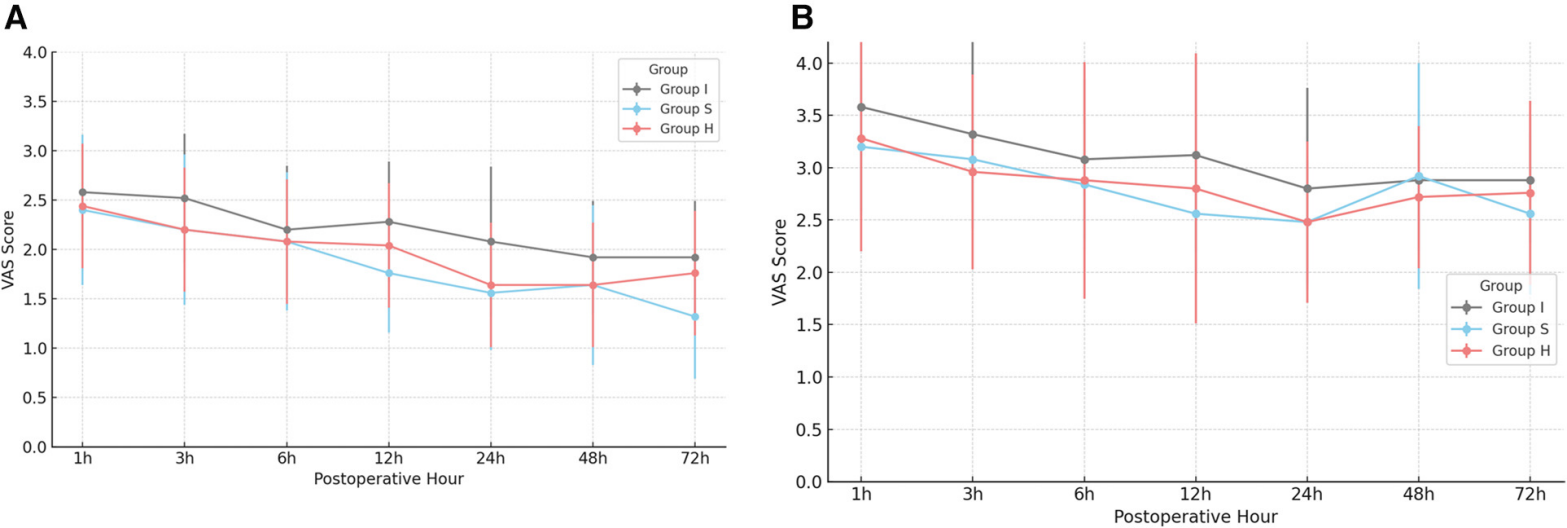
Postoperative resting VAS (VAS-R, VAS-D) pain scores **(A)** postoperative VAS-R scores over time by group. **(B)** Postoperative VAS-D scores over time by group. *Significance, *p* < 0.05, VAS, visual analog scale; VAS-R, resting VAS score; VAS-D, dynamic VAS score.

Postoperative pain was assessed using VAS-R and VAS-D. All groups maintained mean VAS scores below 4 throughout the study period, indicating generally acceptable pain control (Figure 3). Notably, Group S and the Group H demonstrated significantly lower VAS-R scores at 24 and 72 h postoperatively (*p* = 0.01). However, no statistically significant difference in pain scores was observed between Group S and Group H.

**Figure 4:**
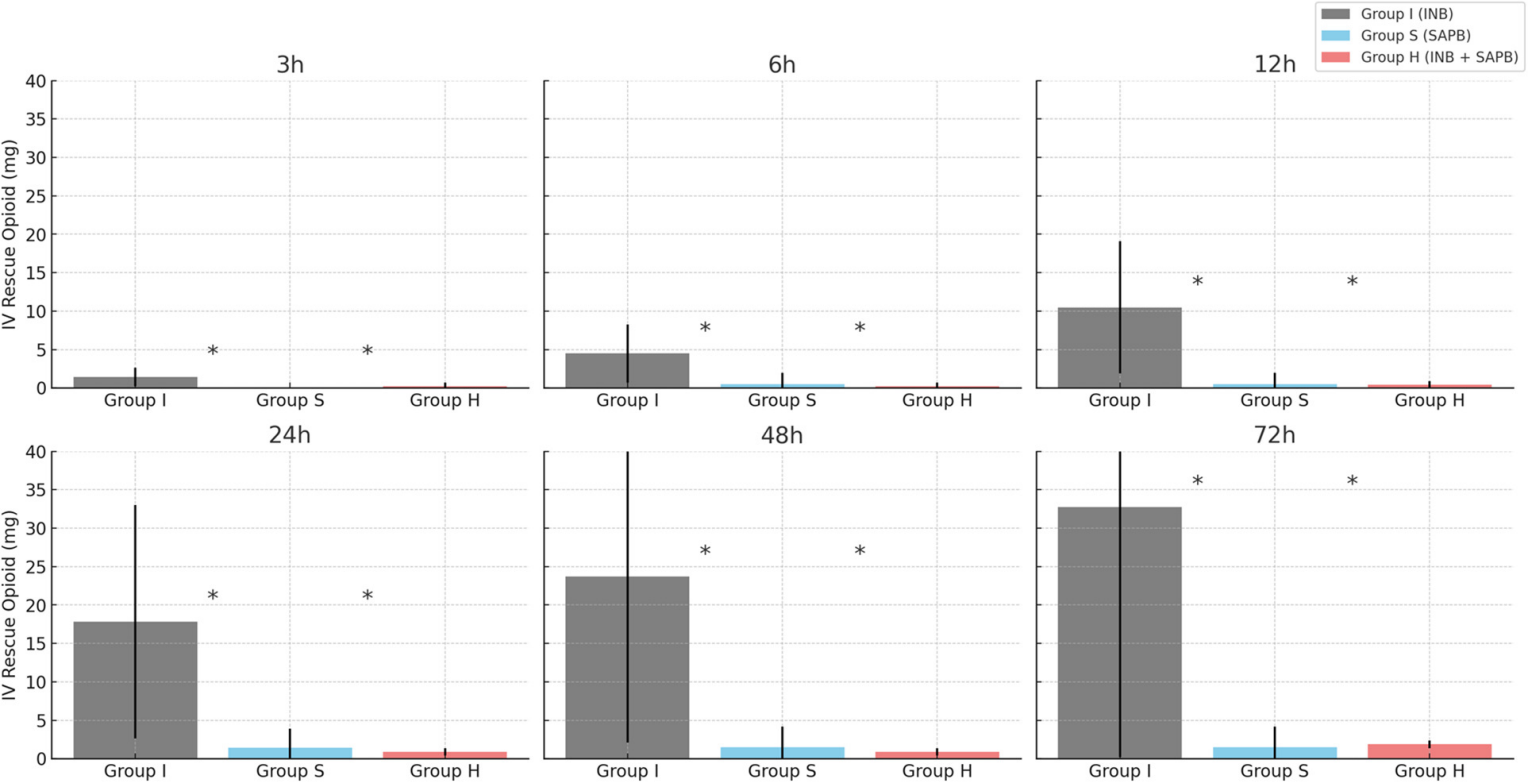
Total postoperative IV rescue opioid consumption by group. *Significance, *p* *<* 0.05; Calculated as MOD (Morphine oral-equivalent dose, mg). IV, intravenous; SD, standard deviation.

Opioid consumption differed significantly among the groups. Group I had significantly higher opioid requirements at all postoperative time points compared to Groups S and H (*p* < 0.01) (Figure 4). Both SAPB-containing groups exhibited similar opioid-sparing effects, with no significant difference between them. No significant differences were observed in postoperative recovery outcomes, including pneumonia incidence (*p* = 0.87), 30-day mortality (*p* = 0.87), chest tube duration (*p* = 0.85), or hospital stay (*p* = 0.65) (Table 2).

## Discussion

Our study demonstrated that continuous SAPB, INB, and combination of SAPB with INB provided optimal pain control following VATS lobectomy. Notably, the addition of INB to continuous SAPB did not confer significant additional analgesic benefit, suggesting that continuous SAPB alone is sufficient for optimal postoperative pain management.

VAS-R and VAS-D scores were significantly lower in the Group S and Group H at 24 h (*p* = 0.01), and 72 h (*p* = 0.01). In contrast, the Group I exhibited higher opioid consumption at all measured postoperative intervals (*p* < 0.01). These results are consistent with previous studies demonstrating that SAPB provides prolonged analgesia and superior postoperative pain control compared to single-injection INB (10, 11). SAPB targets the lateral cutaneous branches of the intercostal nerves (T2–T9), which innervate the lateral thoracic wall, thereby providing effective analgesia of the anterolateral chest wall. Additionally, SAPB may partially block the long thoracic and thoracodorsal nerves, contributing to broader sensory coverage (12–14). SAPB can be performed using two distinct approaches, depending on the plane of local anesthetic injection (15, 16). In this study, the superficial approach was employed.

(1) Superficial SAPB: Local anesthetic is injected into the fascial plane between the SA and LD muscles. This technique primarily blocks the lateral cutaneous branches of the intercostal nerves, providing effective analgesia to the anterolateral chest wall.
(2) Deep SAPB: Local anesthetic is deposited between the serratus anterior muscle and the external intercostal muscles overlying the fourth or fifth rib. This approach enables a broader spread of the anesthetic, potentially allowing for more effective blockade of the intercostal nerves before they divide into terminal branches.

Local anesthetic administered in the serratus plane diffuses both anteriorly and posteriorly, blocking voltage-gated sodium channels and thereby inhibiting nociceptive signal transmission to the spinal cord (12, 13). Unlike thoracic epidural or paravertebral blocks, SAPB does not affect sympathetic pathways, minimizing the risk of hypotension or motor blockade.

While INB remains a commonly employed technique in thoracic surgery, its analgesic effects are often short-lived due to rapid systemic absorption and limited dermatomal spread. In contrast, continuous SAPB offers sustained analgesia, which reduces both breakthrough pain and opioid requirements. These findings are consistent with previous studies demonstrating that SAPB significantly decreases both opioid use and pain scores compared to INB in patients undergoing VATS (17). Although both SAPB and INB have been evaluated individually in thoracic surgical settings, few studies have directly compared continuous SAPB with single-injection INB in the context of VATS lobectomy. Gabriel et al. reported that SAPB more effectively reduced postoperative opioid consumption than INB in patients undergoing VATS wedge resection (18). Other studies have shown that SAPB provides comparable analgesia to TEA with fewer complications, positioning it as a viable first-line alternative (19). A meta-analysis by Liu et al. further reinforced SAPB's role in multimodal analgesia by demonstrating superior pain control compared to both paravertebral and intercostal blocks (20).

Although INB has traditionally been favored by thoracic surgeons due to its simplicity, its limited duration of action makes it less suitable for extended postoperative pain control. In contrast, continuous SAPB provides more stable analgesia during the critical early postoperative period and aligns well with the principles of ERAS by facilitating early mobilization and reducing opioid consumption. The clinical advantages of continuous SAPB include: (1) Prolonged analgesia: Whereas single-injection INB requires frequent opioid rescue medication due to its short duration, continuous SAPB provides stable, sustained pain relief and minimizes opioid-related adverse effects such as nausea, sedation, and respiratory depression. (2) Technical simplicity& safety: SAPB is technically straightforward and avoids complications associated with neuraxial blocks, such as hypotension or motor impairment, common with TEA or PVB (18). (3) Enhanced Patient Recovery: Continuous SAPB supports ERAS goals by enhancing pain control, reducing opioid consumption, promoting pulmonary function, and shortening hospital stays (19).

A unique strength of this study was the deliberate omission of IV PCA allowing an unbiased assessment of the true analgesic efficacy of SAPB and INB without the confounding effect of systemic opioids. In clinical practice, single-injection INB is widely utilized by thoracic surgeons due to its technical simplicity and intraoperative feasibility. However, its short duration of action limits its effectiveness in providing sustained postoperative analgesia. The present study aimed to evaluate continuous SAPB as a practical, surgeon-performed alternative that overcomes this limitation while maintaining procedural simplicity. Furthermore, there is a paucity of studies directly comparing continuous SAPB alone with the combination of SAPB and INB. In our study, the addition of INB to continuous SAPB did not provide additional benefits in terms of postoperative pain control, opioid consumption, or length of hospital stay. This may be explained by overlapping dermatomal coverage, the prolonged analgesic effect of continuous SAPB, and the limited duration of single-injection INB. These results suggest that continuous SAPB alone is sufficient for postoperative pain control following VATS lobectomy, consistent with multimodal analgesia strategies and ERAS objectives. Additionally, SAPB may be considered a suitable alternative to TEA in patients with contraindications to epidural placement (e.g., coagulopathy or hemodynamic instability) while still providing comparable analgesic efficacy.

Despite the strengths of this study, several limitations should be acknowledged. First, this was a single-center trial with a relatively small sample size, which may limit the generalizability of the findings. Larger, multicenter studies are necessary to validate these results. Second, although continuous SAPB has been proposed as a viable alternative to TEA or PVB, our study did not include a control group using these standard techniques. Future randomized trials should directly compare continuous SAPB with TEA or PVB to establish comparative efficacy. Third, future investigations should evaluate variations in SAPB infusion rates, extended infusion durations beyond 72 h, and the use of adjuvant agents (e.g., dexmedetomidine or dexamethasone) to enhance analgesic efficacy and duration. Fourth, we did not separately assess ipsilateral shoulder pain, which can be a relevant component of postoperative discomfort following thoracic surgery. While global pain scores were recorded, future studies should incorporate targeted assessment of referred shoulder pain to more comprehensively evaluate analgesic effectiveness. Fifth, we did not include patients undergoing sublobar resections such as segmentectomy or wedge resection. This decision was made to ensure uniformity in surgical extent and pain expectations across study groups. However, given the increasing clinical adoption of sublobar techniques, future studies should evaluate the applicability and efficacy of SAPB and other regional analgesia methods in these populations.

## Conclusions

This study demonstrates that continuous SAP) provides superior postoperative analgesia and significantly reduces opioid consumption compared to single-injection INB in patients undergoing video-assisted VATS lobectomy. Notably, continuous SAPB alone was as effective as the combination of SAPB and INB, indicating no additional analgesic benefit from combining the two techniques. Given its technical simplicity, sustained analgesic effect, and opioid-sparing advantages, continuous SAPB represents a practical and effective component of multimodal analgesia within ERAS protocols. Further large-scale, multi-center randomized trials comparing continuous SAPB with TEA are warranted to further define its role in postoperative pain management strategies for thoracic surgery.

## Data Availability

The raw data supporting the conclusions of this article will be made available by the authors, without undue reservation.
