# Supplementary material for: Analgesic efficacy of continuous serratus anterior plane block versus intercostal nerve block and their combination in VATS lobectomy: results from a prospective randomized trial
Source: Front Surg. 2025 May 27;12:1607150. doi: 10.3389/fsurg.2025.1607150 (PMC12148914; doi:10.3389/fsurg.2025.1607150)
Supplement: Supplementary file 1 [file Table1.docx]

**Supplement 1.** Postoperative resting VAS (VAS-R, VAS-D) scoring, shown by a group

|  | INB (group I)  (n=30) | SAPB (group S)  (n=30) | INB with SAPB (group H)  (n=30) | *P* value* |
| --- | --- | --- | --- | --- |
| **VAS-R** |  |  |  |  |
| 1h | 2.58 (±0.48) | 2.40 (±0.76) | 2.44 (±0.63) | 0.28 |
| 3h | 2.52 (±0.65) | 2.20 (±0.76) | 2.20 (±0.63) | 0.17 |
| 6 h | 2.20 (±0.65) | 2.08 (±0.70) | 2.08 (±0.63) | 0.80 |
| 12 h | 2.28 (±0.61) | 1.76 (±0.60) | 2.04 (±0.63) | 0.054 |
| 24 h | 2.08 (±0.76) | 1.56 (±0.58) | 1.64 (±0.63) | 0.01 |
| 48 h | 1.92 (±0.57) | 1.64 (±0.81) | 1.64 (±0.63) | 0.21 |
| 72 h | 1.92 (±0.57) | 1.32 (±0.63) | 1.76 (±0.63) | 0.01 |
| **VAS-D** |  |  |  |  |
| 1hr | 3.58 (±0.99) | 3.20 (±0.65) | 3.28 (±1.08) | 0.28 |
| 3hr | 3.32 (±0.90) | 3.08 (±0.40) | 2.96 (±0.93) | 0.26 |
| 6 h | 3.08 (±0.76) | 2.84 (±0.62) | 2.88 (±1.13) | 0.58 |
| 12 h | 3.12 (±0.93) | 2.56 (±0.58) | 2.80 (±1.29) | 0.13 |
| 24 h | 2.80 (±0.96) | 2.48 (±0.59) | 2.48 (±0.77) | 0.26 |
| 48 h | 2.88 (±0.67) | 2.92 (±1.08) | 2.72 (±0.68) | 0.67 |
| 72 h | 2.88 (±0.60) | 2.56 (±0.77) | 2.76 (±0.88) | 0.33 |

*Significance, *p*<0.05
Data expressed as mean (SD)
SD, standard deviation; VAS, Visual Analog Scale; VAS-R, resting VAS score; VAS-D, dynamic VAS score
