# Supplementary material for: Analgesic efficacy of continuous serratus anterior plane block versus intercostal nerve block and their combination in VATS lobectomy: results from a prospective randomized trial
Source: Front Surg. 2025 May 27;12:1607150. doi: 10.3389/fsurg.2025.1607150 (PMC12148914; doi:10.3389/fsurg.2025.1607150)
Supplement: Supplementary file 3 [file Table3.docx]

**Supplement 3.** Total postoperative IV rescue analgesic consumption by group

| Total IV  rescue analgesics,  mg† | | INB (group I)  (n=30) | SAPB (group S)  (n=30) | INB with SAPB (group H)  (n=30) | *P* value* |
| --- | --- | --- | --- | --- | --- |
| 3h | 1.44 (±1.20) | | 0.00 | 0.20 (±0.48) | <0.01 |
| 6 h | 4.47 (±3.75) | | 0.50 (±1.44) | 0.20 (±0.48) | <0.01 |
| 12 h | 10.49 (±8.61) | | 0.50 (±1.44) | 0.40 (±0.48) | <0.01 |
| 24 h | 17.83 (±15.22) | | 1.40 (±2.51) | 0.90 (±0.48) | <0.01 |
| 48 h | 23.69 (±21.58) | | 1.50 (±2.70) | 0.90 (±0.48) | <0.01 |
| 72h | 32.78 (±32.58) | | 1.50 (±2.70) | 1.86(±0.48) | <0.01 |

*Significance, *p<*0.05
†Calculated as MOD
Data expressed as mean (SD)
IV, intravenous; MOD, morphine oral-equivalent dose; SD, standard deviation
