# Supplementary material for: Analgesic efficacy of continuous serratus anterior plane block versus intercostal nerve block and their combination in VATS lobectomy: results from a prospective randomized trial
Source: Front Surg. 2025 May 27;12:1607150. doi: 10.3389/fsurg.2025.1607150 (PMC12148914; doi:10.3389/fsurg.2025.1607150)
Supplement: Supplementary file 4 [file Table4.docx]

**Supplement 4.** Post-hoc comparison of total postoperative IV rescue analgesic consumption by group

| Total IV  rescue analgesics,  mg† | | SAPB  (Group S)  n=30 | INB (Group I)  n=30 | p-value  (Group S vs. Group I) | SAPB+INB (Group H)  n=30 | p-value  (Group S vs. Group H) |
| --- | --- | --- | --- | --- | --- | --- |
| 3h | 0.00 | | 1.44 (±1.20) | <0.01 | 0.20 (±0.48) | 0.15 |
| 6h | 0.50 (±1.44) | | 4.47 (±3.75) | <0.01 | 0.20 (±0.48) | 0.56 |
| 12h | 0.50 (±1.44) | | 10.49 (±8.61) | <0.01 | 0.40 (±0.48) | 0.68 |
| 24h | 1.40 (±2.51) | | 17.83 (±15.22) | <0.01 | 0.90 (±0.48) | 0.44 |
| 48h | 1.50 (±2.70) | | 23.69 (±21.58) | <0.01 | 0.90 (±0.48) | 0.35 |
| 72h | 1.50 (±2.70) | | 32.78 (±32.58) | <0.01 | 1.86 (±0.48) | 0.72 |

*Significance, *p<*0.05
†Calculated as MOD
Data expressed as mean (SD)
IV, intravenous; MOD, morphine oral-equivalent dose; SD, standard deviation
